# Pyramidal cells in V1 of African rodents are bigger, more branched and more spiny than those in primates

**DOI:** 10.3389/fnana.2014.00004

**Published:** 2014-02-10

**Authors:** Guy N. Elston, Paul Manger

**Affiliations:** ^1^Centre for Cognitive NeuroscienceSunshine Coast, QLD, Australia; ^2^School of Anatomical Sciences, Faculty of Health Sciences, University of the WitwatersrandJohannesburg, South Africa

**Keywords:** striate, cortex, dendrite, spine, Lucifer yellow

## Abstract

Pyramidal cells are characterized by markedly different sized dendritic trees, branching patterns, and spine density across the cortical mantle. Moreover, pyramidal cells have been shown to differ in structure among homologous cortical areas in different species; however, most of these studies have been conducted in primates. Whilst pyramidal cells have been quantified in a few cortical areas in some other species there are, as yet, no uniform comparative data on pyramidal cell structure in a homologous cortical area among species in different Orders. Here we studied layer III pyramidal cells in V1 of three species of rodents, the greater cane rat, highveld gerbil, and four-striped mouse, by the same methodology used to sample data from layer III pyramidal cells in primates. The data reveal markedly different trends between rodents and primates: there is an appreciable increase in the size, branching complexity, and number of spines in the dendritic trees of pyramidal cells with increasing size of V1 in the brain in rodents, whereas there is relatively little difference in primates. Moreover, pyramidal cells in rodents are larger, more branched and more spinous than those in primates. For example, the dendritic trees of pyramidal cells in V1 of the adult cane rat are nearly three times larger, and have more than 10 times the number of spines in their basal dendritic trees, than those in V1 of the adult macaque (7900 and 600, respectively), which has a V1 40 times the size that of the cane rat. It remains to be determined to what extent these differences may result from development or reflect evolutionary and/or processing specializations.

## Introduction

Studies in primates have revealed marked differences in pyramidal cell structure among different cortical areas (Lund et al., 1993; Elston, 2002, 2003a; Jacobs and Scheibel, 2002; Bianchi et al., 2012). Cells tend to become larger, more branched, and more spiny with progression through cortical areas associated with primary sensory, sensory association, and executive function (see Elston, 2002, 2003a; Jacobs and Scheibel, 2002; Spruston, 2008; Defelipe, 2011; for reviews). The extent of these interspecies differences varies according to the cortical area studied. For example, there is relatively little difference in the size, branching structure, and number of spines (putative excitatory inputs) in the dendritic trees of pyramidal cells in V1 among primates, whereas cells in the granular prefrontal cortex (gPFC) are progressively larger, more branched and more spinous the larger the gPFC (Elston et al., 2006b). Those in human gPFC are, on average, 23-fold more spinous than those in V1 of the macaque; however, it remains to be determined to what extent pyramidal cells may differ among cortical areas in other non-primate mammalian species.

Quantification of pyramidal cells in rodents has also revealed regional specialization in the size, branching structure, and spine density of their dendritic trees. Those in the mouse have been shown to differ among visual, motor and somatosensory cortex (Ballesteros-Yáñez et al., 2006; Benavides-Piccione et al., 2006). In the South American rodent, the cutia, as in primates, cells become larger, more branched, and more spinous with increasing distance from primary visual cortex (Elston et al., 2006c); however, the extent of these differences remain unknown.

Neurons in VI of rodents show orientation preference (Tiao and Blakemore, 1976; Mangini and Pearlman, 1980; Metin et al., 1988; Girman et al., 1999; Schuett et al., 2002; Van Hooser et al., 2005); but, rodent V1 lacks the orientation maps and ocular dominance columns typically associated with primate V1 (e.g., Hubel and Wiesel, 1968; Van Hooser et al., 2005). Furthermore, rodents, while having distinct visuotopic maps, are thought to have fewer clearly differentiated visual cortical areas than primates and have a substantially smaller V1 than primates (Felleman and Van Essen, 1991; Rosa and Krubitzer, 1999; Van Hooser et al., 2005). Variability in the neuronal composition of V1 has also been reported among rodents (Campi et al., 2011). Thus, the question becomes, what, if any, specializations in cortical microcircuitry underpin the differences in visual processing in rodents and primates, and how do they relate to neuronal composition and brain size?

Here we quantified layer III pyramidal cell structure in V1 of the greater cane rat, the bushveld gerbil and the four-striped mouse and compared them with previously published data sampled from the baboon, macaque monkey, vervet monkey, owl monkey, marmoset, galago, and tree shrew. We found that, irrespective of brain size, pyramidal cells in V1 of rodents are larger, more branched and more spinous than those in primates.

## Methods

Three African rodent species, including the greater cane rat (*Thryonomys swinderianus*), the bushveld gerbil (*Tatera branstii*), and the four-striped mouse (*Rhabdomys pumilio*) were included for study. The rodents used in the present study were caught from wild populations in South Africa with permission and supervision from the appropriate wildlife directorates. All animals (5 cane rat, 5 striped mice, 6 gerbil) were treated and used according to the guidelines of the University of the Witwatersrand Animal Ethics Committee, which parallel those of the NIH for the care and use of animals in scientific experimentation. Although the ages of the animals is not known, the body mass and physical development indicated that they were sexually mature adults. Animals were sedated with an I.M. injection of ketamine hydrochloride (40 mg/kg) and xylazine hydrochloride (4 mg/kg) and overdosed by I.P. injection of sodium pentobarbital (100 mg/kg).

**Figure 1:**
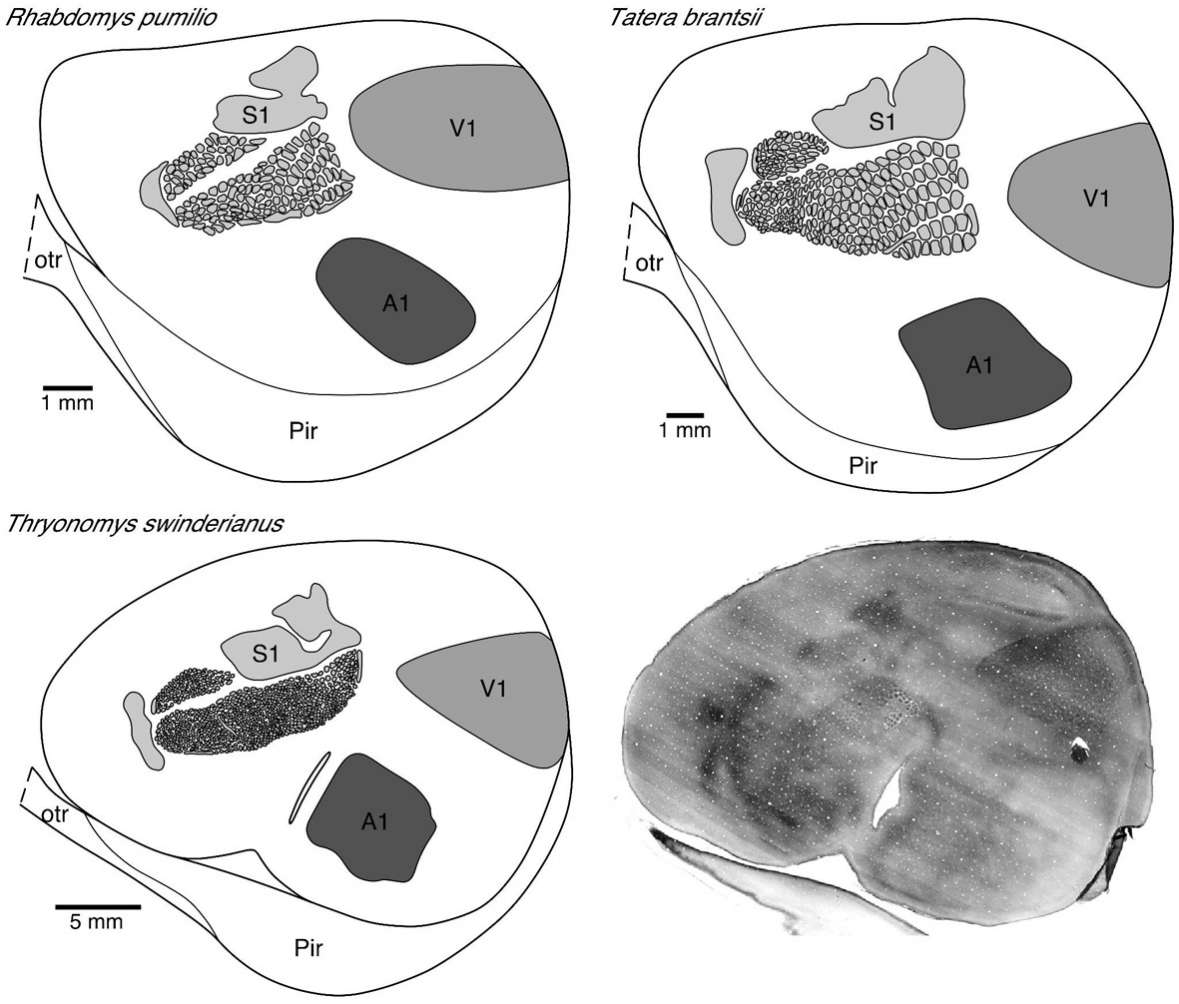
**Photomicrograph (bottom right) of a tangential section cut from a “flat-mount” preparation of the entire left hemisphere of the greater cane rat that was processed for cytochrome oxidase reactivity.** Note the easily distinguishable V1 located in the dorsocaudal region with sharply delineated boundaries. Note also the barrels in the primary somatosensory cortex.

Methodology used in the present investigation was exactly the same as used in our previous cell injection studies. Animals were perfused transcardially with phosphate buffered saline (0.95% saline in 0.1 M phosphate buffer [PB; pH 7.2]) then paraformaldehyde (4% in PB). The brains were removed and the right hemispheres were flat-mounted (see Elston and Rosa, 1997, for details). The flattened hemispheres were left overnight in 4% paraformaldehyde in 0.1 M PB at 4°C. Alternate serial 250 and 50 μm sections were then cut tangential to the cortical surface with the aid of a Vibratome. The 50 μm tangential sections were processed for cytochrome oxidase according to the Wong-Riley method (Wong-Riley, 1979) to reveal the size and location of V1 and other primary cortical areas (Figure 1). Other tangential sections were pre-labeled with 4,6 diamidino-2-phenylindole (10^−5^ mol/L, D9542, Sigma, USA) for approximately 10 min at room temperature. These sections were then mounted into a perspex chamber on a fixed stage fluorescence microscope (Zeiss Axioskop II Plus) and individual cells were injected with Lucifer Yellow (LY; L-0259, Sigma: 8% in 0.1 M Tris buffer, pH 7.4).

**Figure 2:**
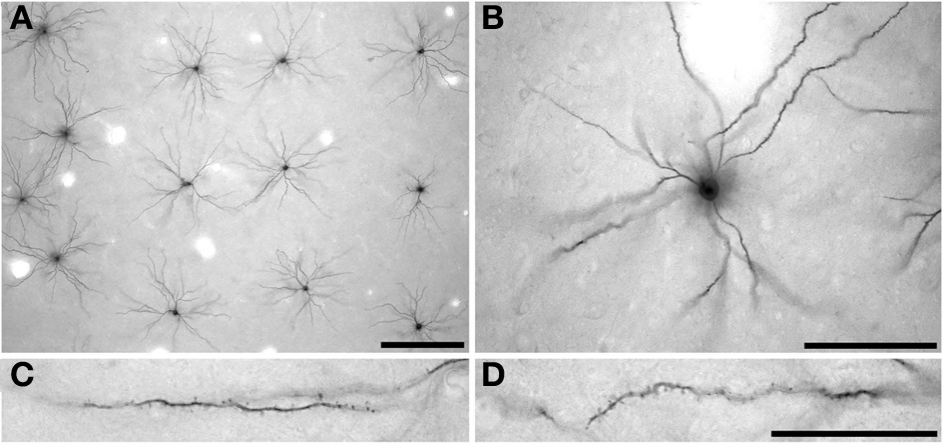
**Low- (A) intermediate- (B) and high- (C,D) power photomicrographs of layer III pyramidal cells in primary visual cortex of the greater cane rat (*Thryonomys swinderianus*) that were injected with Lucifer Yellow and processed for a DAB (3,3′-diaminobenzidine) reaction product.** Cells were injected in a grid-like pattern **(A)**, with each cell being sufficiently spaced from other to avoid cluttering of dendrites **(B)**. The apical dendrite is observed at low and intermediate power in tangential sections (**A,B**, respectively) as a black dot over the cell body, due to the extra depth of DAB precipitate in the issuing process as compared with the surrounding cell body. **(C)** and **(D)** indicate the spine density along the basal dendrites of the layer III pyramidal cells, where the cell body is to the right and the distal tips of the dendrites to the left. Scale bar in **(A)** =250 μm, scale bar in **(B)** =100 μm, and the scale bar in **(D)** =50 μm and applies to **(C)** and **(D)**.

Intracellular injection was by continuous negative current. Once approximately 50 cells had been injected in each slice, the sections were processed with anti-LY (1:400 000) in stock solution (2% bovine serum albumin [Sigma A3425], 1% Triton X-100 {BDH 30632}, 5% sucrose in 0.1 mol/l PB) for 5 days at room temperature. 3,3′-diaminobenzidine (DAB; Sigma D 8001) was used as the chromogen (Figure 2) (see Elston et al., 1997, for details). Three hundred and twenty pyramidal neurons were injected in the present study. Two hundred and thirty-three of these cells were included for analyses as they had an unambiguous apical dendrite, issuing from the upper cell body and projecting directly toward the viewer, were well filled, and had their entire basal dendritic trees contained within the slice (108 cells in the cane rat, 61 cells in the gerbil, and 62 cells in the striped mouse). When viewed in the tangential plane the basal dendritic trees of pyramidal cells have a roughly spherical dendritic tree (with some variation) as opposed to the pyramid shape observed in transverse sections. The apical dendrite is observed at low and intermediate power in tangential sections as a black dot over the cell body, due to the extra depth of DAB precipitate in the issuing dendrite as compared with the surrounding cell body (see Figures 2A,B). Moreover, the trajectory of the apical dendrite can be viewed at high power by changing the plane of focus, as can its diameter compared with the basal dendrites issuing from the under side of the cell body. Only neurons that we could establish had their cell bodies located at the base of layer III were included for analyses (e.g., see Figure 3 of Elston and Rosa, 1997). Moreover, we only included cells from the cases in which we injected pyramidal cells in the central 30 degrees of the visual field to minimize any potential confound attributable to intra-areal variation in pyramidal cell structure in V1 according to visuotopy (e.g., Freire et al., 2010).

Pyramidal cells were drawn with the aid of a Zeiss Axioskop 40 equipped with a camera lucida. Dendritic tree size (the area contained within a polygon joining the outermost distal tips of the basal dendrites) and somal size were determined in 2-dimensions with the aid of NIH-Image software (NIH, Bethesda, US) (e.g., Elston and Rosa, 1997). Branching patterns were determined by performing a Sholl analysis on the 2D drawings of cells (e.g., Sholl, 1953). Spines were drawn along the entire dendrite from the cell body to the distal tip of twenty different dendrites in each species while viewing the cells under a ×100 oil objective, changing focus through the depth of the tissue. Spine density was determined by counting the number of spines per 10 μm length of dendrite as a function of distance from the cell body (e.g., Eayrs and Goodhead, 1959; Valverde, 1967). An estimate of the total number of spines found in the basal dendritic tree of the “average” pyramidal cell in each cortical area was calculated by multiplying the average number of spines in each successive portion of dendrite by the average number of branches for the corresponding region, over the entire dendritic tree (see Figure 2 of Elston, 2001).

Statistical analysis of the dendritic tree size, branching structure, spine density, and cell body size was performed with SPSS, as per our previous studies (e.g., Elston et al., 2005a,b,c,d,e). In particular, One-Way ANOVAS were applied for dendritic tree size and cell body size, repeated-measures ANOVAS were applied to branching complexity and spine density. Tests for differences in slopes and elevations of plots of the dendritic tree size, branching structure, spine density, and cell body size of the present data sampled in rodents and those data previously sampled from primates (Elston and Rosa, 1997, 1998; Elston et al., 1999, 2005a,b,c,d,e, 2006a; Elston, 2003b) was performed with SMATR software (version 2.0) as per Warton et al. (2006).

## Results

The primary visual area was readily identified in “flat-mounts” prepared from the cortical hemispheres and processed for cytochrome oxidase (Figure 1). The absolute size of V1 in which we injected neurons in all species included for study was quantified. All analyses were performed on the left hemisphere. We found that V1 in the greater cane rat (44.09 mm^2^) was larger than that in the bushveld gerbil (14.63 mm^2^), which was larger than that in the four-striped mouse (11.58 mm^2^).

### Basal dendritic tree size

**Figure 3:**
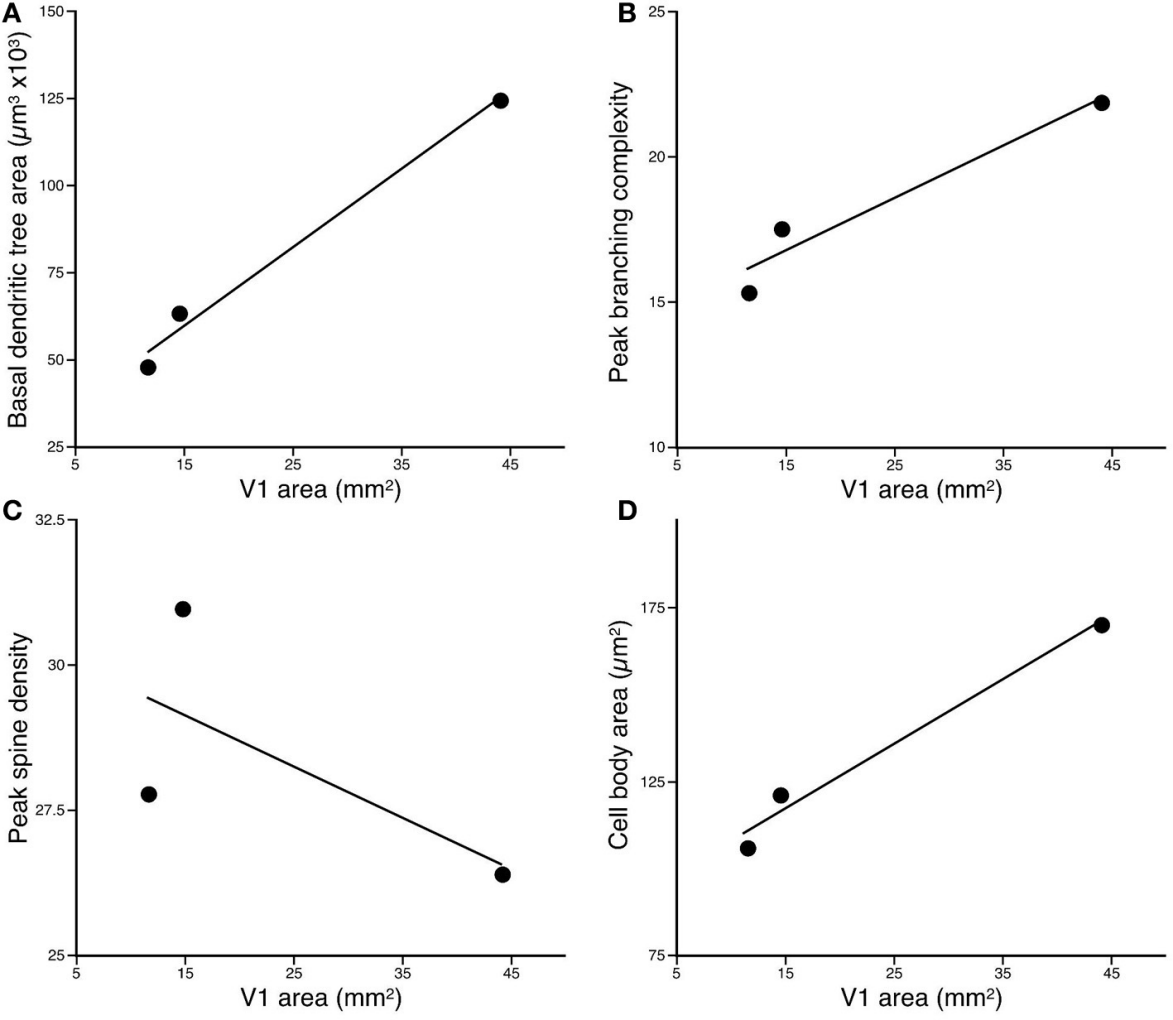
**Plots of (A) the size, (B) peak branching complexity, and (C) peak spine density, vs. the size of V1 of the basal dendritic trees of layer III pyramidal neurons sampled in the primary visual area of the Cane Rat, Bushvelt Gerbil, and Striped Mouse. (D) Plots of cell body size of layer III pyramidal neurons vs. the size of V1.** The size of the dendritic trees (in the tangential plane) was determined by calculating the area contained within a convex hull, which joined the outermost distal dendrites. The branching complexity was determined by counting the number of intersections of the basal dendrites with a series of concentric circles of progressively larger diameter (Sholl analysis). The peak branching complexity was the greatest value obtained in each species. Spine density was calculated by drawing horizontally-projecting basal dendrites of different cells in each case at high power (×100 oil immersion lens) and counting the number of spines per 10 μm interval progressing along the dendrite from the cell body to the distal tips of the dendrite. The peak spine density was the greatest value obtained. Cell body size was determined at high power (×100 oil immersion lens) by tracing around the outer extent of the cell body while focusing through the depth of the soma.

The basal dendritic trees of layer III pyramidal cells in the greater cane rat (*n* = 108, mean ± *SD*: 123.15 ± 24.90 × 10^3^ mm^2^) were larger than those in the bushveld gerbil (*n* = 61, 63.13 ± 16.28 × 103 mm^2^) which, in turn, were larger than those in the four-striped mouse Figure 3 (*n* = 62, 38.55 ± 12.80 × 10^3^ mm^2^; Figure 3). One-Way ANOVAs revealed these differences to be significant [*F*_(2)_ = 393.52, *p* < 0.001]. *Post-hoc* Scheffe tests revealed significant differences among all three species (*p* < 0.001).

### Complexity of the basal dendritic trees

Plots of the results of Sholl analysis in which we counted the number of dendritic intersections in successive concentric circles with radii of 25 μm increments revealed that the peak branching density in the basal dendritic trees of layer III pyramidal cells in the greater cane rat (mean ± SE: 21.99 ± 5.77) was greater than that in the bushveld gerbil (17.43 ± 4.12), which, in turn, was greater than that in the four-striped mouse (15.26 ± 4.05; Figure 3). A repeated measures ANOVA revealed a significant difference in the complexity of the basal dendritic trees between animals [*F*_(2, 228)_ = 167.66, *p* < 0.001]. *Post-hoc* Scheffe tests revealed all species comparisons to be significantly different.

### Spine densities of the basal dendrites

A total of 6484 spines were drawn and tallied. As reported previously (see Elston and DeFelipe, 2002, for review), the spine density along the basal dendrites varied as a function of distance from the cell body to the distal tips. The peak spine density in the basal dendritic trees of layer III pyramidal cells in the greater cane rat (*n* = 10; mean ± SE: 26.4 ± 3.66) was less than that in the bushveld gerbil (*n* = 2; 31.0 ± 3.03), which was greater than that in the four-striped mouse (*n* = 10; 27.9 ± 1.81; Figure 3). A repeated measures ANOVA revealed a significant difference in the spine density between animals [*F*_(2, 19)_ = 10.18, *p* = 0.001]. *Post-hoc* Scheffe tests revealed all species comparisons to be significantly different.

By combining data from the Sholl analyses and spine density counts we calculated an estimate of the total number of dendritic spines in the basal dendritic tree of the “average” layer III pyramidal neuron in V1 of each species. The total number of spines in the basal dendritic trees of layer III pyramidal cells in the greater cane rat (7903) was greater than that in the bushveld gerbil (4316), which, in turn, was greater than that in the four-striped mouse (2141).

### Cell bodies

The cell bodies of layer III pyramidal cells in the greater cane rat (mean ± *SD*: *n* = 108, 170.71 ± 19.84 mm^2^) were larger than those in the bushveld gerbil at (*n* = 61, 121.36 ± 23.43 mm^2^), which, in turn, were larger than those in the four-striped mouse Figure 3. (*n* = 62, 107.07 ± 15.86 mm^2^; Figure 3). One-Way ANOVAs revealed these differences to be significant [*F*_(2)_ = 239.75, *p* < 0.001]. *Post-hoc* Scheffe tests revealed significant differences among all three species (*p* < 0.001).

## Discussion

Here we studied the structure of layer III pyramidal cells in the primary visual cortex of three African rodents, the greater cane rat, the bushveld gerbil, and the four-striped mouse. We present four main findings: (1) the absolute size of the cerebral cortex occupied by V1 differs up to 4-fold among the three species; (2) there are differences in the size, branching structure, spine density, and total number of spines in the basal dendritic trees of layer III pyramidal cells in V1; (3) the spine density may vary independently of the size and branching structure of the dendritic trees among species; and (4) there is a trend between the size and number of spines in the dendritic trees of pyramidal cells and the size of V1 such that the larger V1 the larger and more spinous are the cells.

**Figure 4:**
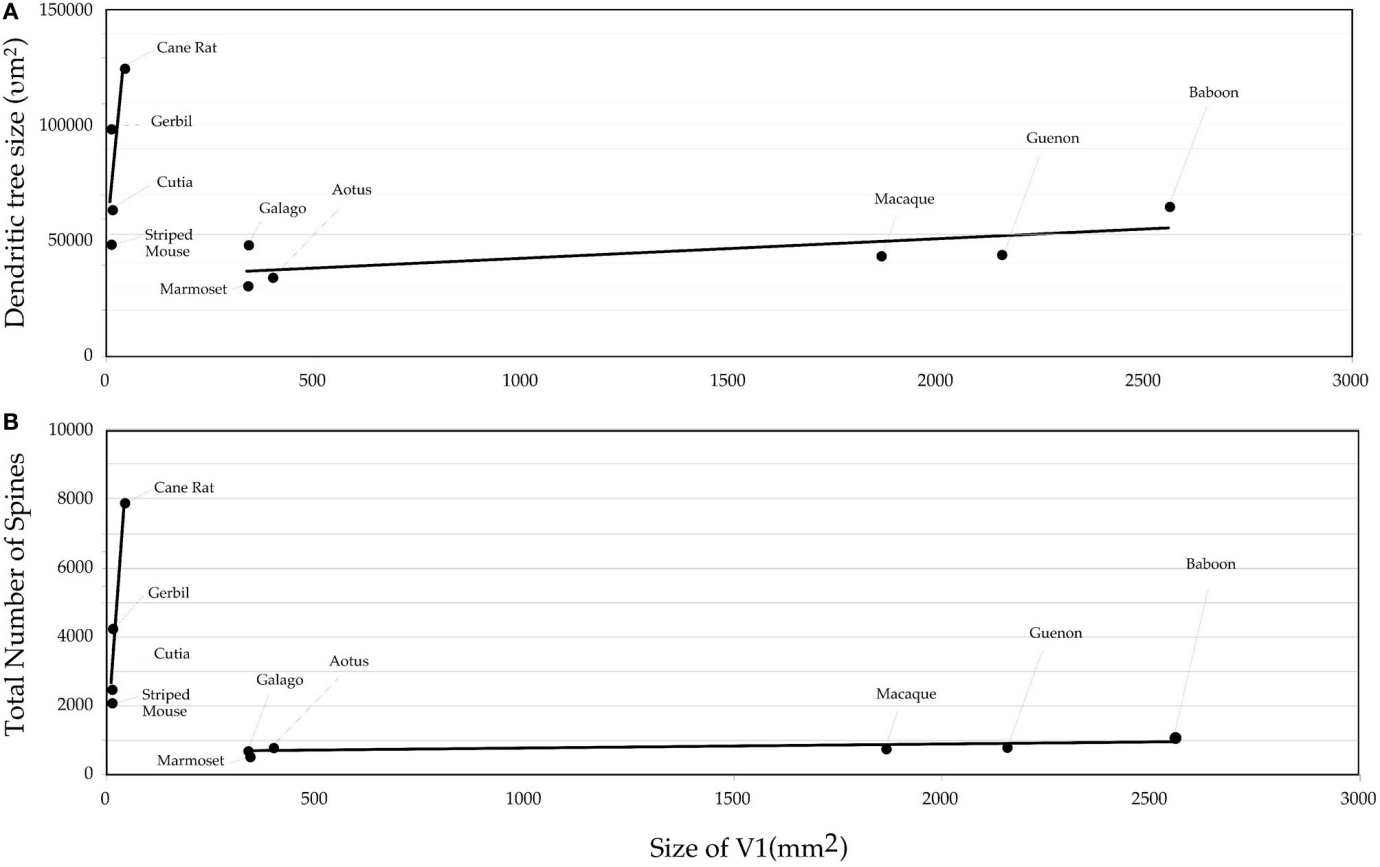
**Graph of the (A) size and (B) the number of dendritic spines in the “average” pyramidal cell vs. the size of the primary visual area (V1) in the cane rat, bushvelt gerbil, striped mouse, agouti, tree shrew, galago, marmoset monkey, owl monkey, guenon (vervet monkey), macaque monkey, and baboon.** Separate trend lines (linear regressions) are illustrated for the rodent and primate data.

Comparison of these data obtained from African rodents with those sampled by the same methodology from V1 of other species such as the South American rodent cutia (Elston et al., 2006c), the archontan tree shrew (Elston et al., 2005c), and primates (galago, Elston et al., 2005b; marmoset, Elston et al., 1999; owl monkey, Elston, 2003b; vervet monkey, Elston et al., 2005d; macaque monkey, Elston and Rosa, 1997, 1998; and baboon, Elston et al., 2005e) reveal marked differences in the neuron structure and brain size between primate and rodent species (Table 1, Figure 4). Whereas in rodents the basal dendritic trees of pyramidal cells are increasingly larger in species in which V1 is increasingly larger, there is relatively little difference in the basal dendritic tree size of pyramidal cells in V1 in primates. Moreover, in rodents pyramidal cells are increasingly more spinous and branched in species in which V1 is increasingly larger in size, whereas there is relatively little difference in the number of spines and branches in pyramidal cells in V1 in primates. Possible developmental and evolutionary influences are discussed below.

**Table 1:** **Data on the size of the brain and V1, and the size, branching complexity, spine density, and total number of spines in the dendritic trees of layer III pyramidal cells in species included in the present investigation**.

**Species**	**Brain mass (g)**	**Area of V1 (mm^2^)**	**Number of cells included in analyses**	**Basal dendritic tree area (μm^2^)**	**Peak branching complexity**	**Peak spine density**	**Total number of spines**
Striped mouse	0.67^a^	11.58^a^	62^a^	48.5^a^	15.3^a^	27.9^a^	2141^a^
Highveld gerbil	1.61^a^	14.63^a^	61^a^	63.3^a^	17.5^a^	31.0^a^	4316^a^
Cane rat	12.9^a^	44.09^a^	108^a^	124.4^a^	21.9^a^	24.6^a^	7903^a^
Cutia	19.45^b^	14.07^b^	90^b^	98.3^b^	21.6^b^	11.9^b^	2524^b^
Tree shrew	3.2^c^	30.85^d^	50^d^	63.4^d^	22.8	11.1	1507^d^
Galago	10.3^c^	343^e^	45^e^	48.4^e^	18.0^e^	6.3^e^	556^e^
Marmoset	7.7^c^	341^f^	25^f^	30.6^f^	15.9^f^	7.7^f^	699^f^
Owl monkey	17.1^c^	400^g^	22^g^	34.4^g^	16.9^g^	9.9^g^	773^g^
Macaque monkey	70.8^c^	1866^h^	213^h^	43.6^h^	16.6^h^	6.9^h^	734^h^
Vervet monkey	72.6^c^	2156^i^	81^i^	44.3^i^	18.5^i^	7.4^i^	795^i^
Chacma baboon	181^c^	2559^j^	84^j^	65.0^j^	20.9^j^	8.1^j^	1077^j^

a Present study

b Elston et al., 2006a

c Stephan et al., 1981

d Elston et al., 2005c

e Elston et al., 2005b

f Elston et al., 1999

g Elston, 2003b

h Elston and Rosa, 1997, 1998; Elston et al., 2005a

i Elston et al., 2005d

j Elston et al., 2005e

### Developmental influences on the mature pyramidal cell phenotype

Developmental studies in the macaque monkey reveal that the dendritic trees of layer III pyramidal cells at birth are larger than those observed in the adult four-striped mouse and bushveld gerbil, but then become smaller during maturation into adulthood (Elston et al., 2010). In addition, pyramidal cells in V1 of the 3 1/2 month old macaque monkey, the time of peak synaptic exuberance (Rakic et al., 1986; Bourgeois and Rakic, 1993; Bourgeois et al., 1994), are considerably more spinous than those in the adult four-striped mouse and bushveld gerbil (Elston et al., 2010). It is natural to ask then whether the differences observed in pyramidal cell structure in V1 of the mature brain of rodents and primates may arise from different growth profiles. While any such differences in the growth profile of pyramidal cells may conceivably account for the differences reported in V1 of the adult macaque monkey and the four-striped mouse, bushveld gerbil, and cutia, they cannot account for the differences observed between the macaque monkey and the greater cane rat. The basal dendritic trees of layer III pyramidal cells in the adult greater cane rat are more than twice the size (122.85 × 10^3^ μm^2^) and approximately twice as spinous (7903 spines) as the largest, most spiny cells observed in the macaque during development (53.93 × 10^3^ μm^2^ and 3900 spines, Elston et al., 2010). The basal dendritic trees of layer III pyramidal cells in the adult gerbil are larger than those observed in the macaque at any age. Furthermore, the basal dendritic trees of layer III pyramidal cells in V1 of the adult greater cane rat, gerbil, and striped mouse are larger than those observed in V1 of the developing and adult marmoset monkey (Oga et al., 2013) Thus, while some of the differences in pyramidal cell structure reported here between adult rodents and primates may be attributable to different growth profiles, they cannot be fully accounted for by development alone.

### Phylogenetic differences in the mature pyramidal cell phenotype

**Figure 5:**
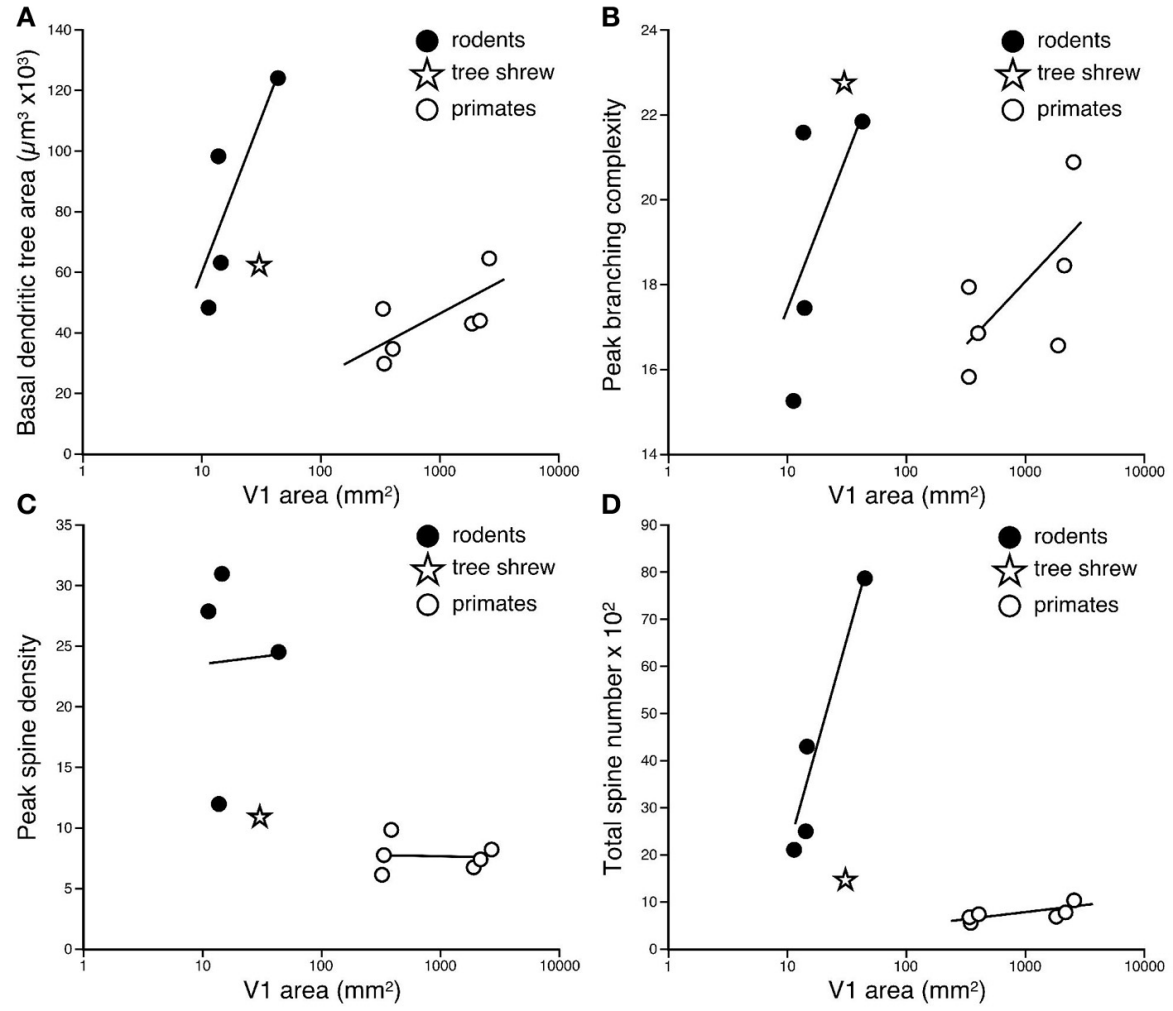
**Logarhythmic plots of the basal dendritic tree areas (A), peak branching complexity (B), peak spine density (C), and total spine number (D) of the “average” pyramidal cell against the size of the primary visual area (V1) in the cane rat, bushveld gerbil, four-striped mouse, agouti, tree shrew, galago, marmoset monkey, owl monkey, guenon (vervet monkey), macaque monkey, and baboon (see Table 1 for raw data and sources of data).** Separate trend lines (linear regressions) are illustrated for the rodent and primate data. Tests for differences in slopes and elevations (using the software SMATR ver 2.0, Warton et al., 2006) between the linear correlations of the rodent and primate data were all statistically significant (*p* < 0.05). Note that in all comparisons, the data for the tree shrew is more closely aligned with the rodent data, rather than the primate data.

**Figure 6:**
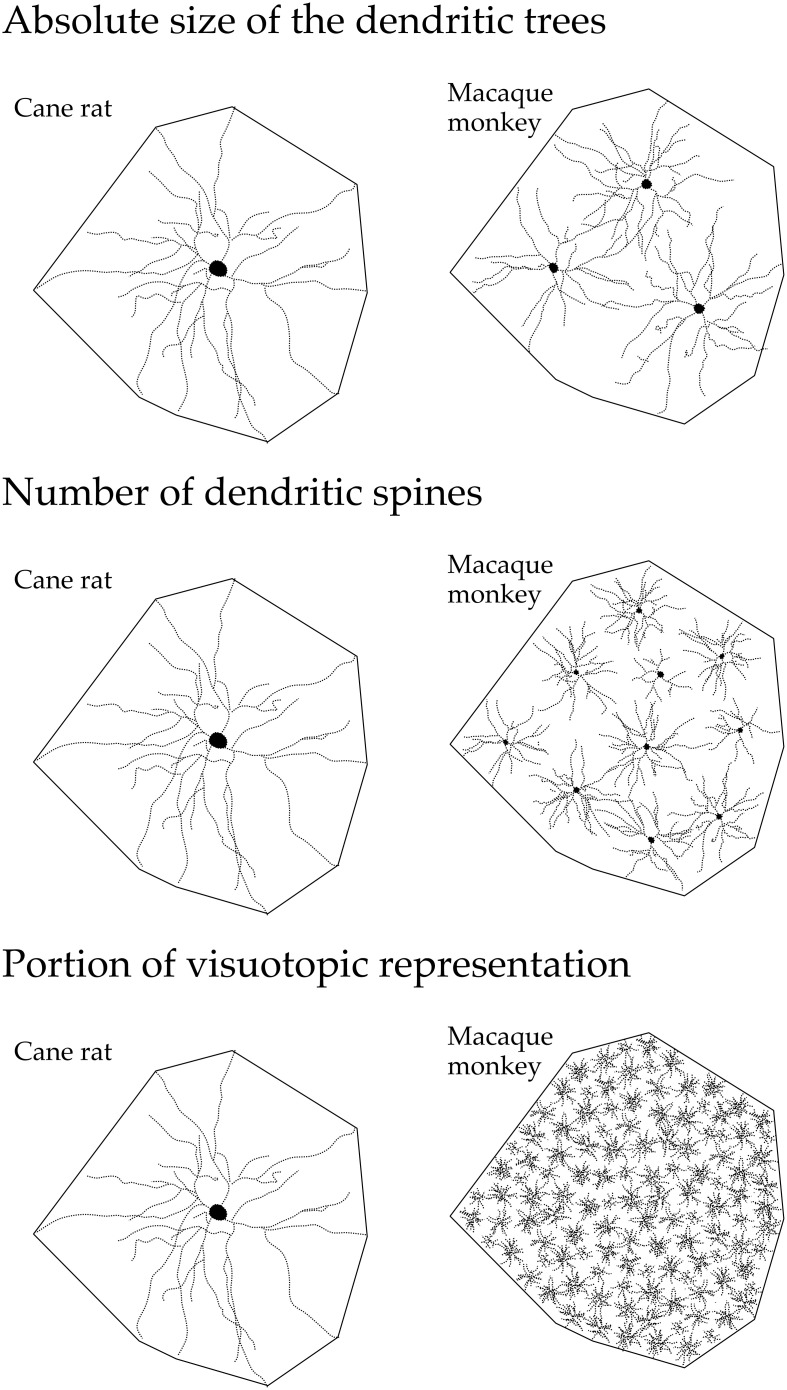
**Line drawings, not drawn to scale, illustrating (top) the “average” dendritic tree of layer III pyramidal cells in V1 of the adult cane rat is nearly three times the size of that in the adult macaque monkey.** Differences in the size, branching structure, and spine density result in a more than 10-fold difference (middle) in the estimate of the total number of spines, putative excitatory inputs, in the basal dendritic tree of the “average” layer III pyramidal cell between the two species. As V1 in the adult macaque is, on average, more than 40 times the size of V1 in the adult cane rat (bottom), the dendritic trees of cells in the adult cane rat sample a portion of the visuotopic field approximately 120 times larger than do those in the adult monkey (i.e., the dendritic trees of cells in the cane rat are three times larger than those in the macaque and sample from a visuotopic representation 40 times smaller than do those in the macaque). Thus, 120 cells are required in the macaque monkey V1 to achieve the spatial coverage of a single cell in the cane rat. One hundred and twenty cells in the adult macaque V1 have, on average, a sum total of more than 88,000 spines (120 × 743), being more than 110 times the number of spines in an individual cell in the adult cane rat. Although this is a fairly simplistic calculation, and doesn't take into account features such as branching density (space filling), neuron density, and reciprocal connectivity, it does, we hope, reveal that cortical circuitry in V1 of the cane rat is quantifiably different to that in the macaque monkey.

The difference in the relationship in rodents and primates between the size of pyramidal cells and the size of V1 could hardly be more dramatic (Figure 4). Statistical analyses revealed significant differences in the slopes and elevations of the regressions between rodents and primates for all parameters tested (Figure 5; see Warton et al., 2006). It appears likely that different principles determine pyramidal cell structure in V1 of adult rodents and primates, which cannot be attributed to a simple cross-species scaling algorithm. For example, the basal dendritic field areas of pyramidal cells in V1 of the greater cane rat are, on average, nearly three times larger than those in the macaque monkey, while the brain of the greater cane rat weighs only 20% that of the macaque monkey and the size of V1 in the cane rat is 2.5% that of the macaque. Moreover, pyramidal neurons in V1 of all rodents included for analyses have considerably more spines in their dendritic trees than those in V1 of primates: those in the cane rat have more than 10 times the number of spines than do those in the macaque monkey (Figure 6).

Previously it has been suggested that the dendritic trees of pyramidal cells may be larger and more branched, may be smaller and less branched, or be the same, in species with increasingly larger cerebral cortex (see Figure 37 of Elston, 2007). Moreover, it has been speculated that pyramidal neurons in species characterized by similar sized cortices may have dramatically different dendritic trees (see Ringo, 1991; Elston, 2007, for reviews). The present data confirms that, within V1, pyramidal cells in small brains may be larger and more branched and more spinous than those in large brains. It then becomes natural to ask why the relationship between the size/branching structure of pyramidal cells and the size of V1/the brain in rodents differs so dramatically to that in primates.

One possibility is that the data reflect fundamentally different evolutionary principles in rodents and primates (see Gould, 2002; Manger, 2005a,b for reviews). In V1 in rodents, the larger the brain, the larger, more branched, and more spinous the neurons, as compared with smaller brains. In V1 in primates, the size, branching complexity, and number of spines is relatively constant among pyramidal cells irrespective of the size of the brain. It could be argued, for example, that mechanisms that determine cortical size and neuron size are linked in rodents but not primates. However, if this were a feature of phylogeny one might expect to see a similar trend in other cortical areas. There are as yet no comparative data of this type in sensory areas such as somatosensory cortex or auditory cortex. There are, however, data sampled from gPFC in primates of different sized brains. In gPFC in primates, pyramidal cells are progressively larger, more branched and more spinous in species with increasing size of the granular PFC (Elston et al., 2006b). That is to say, while there is little difference in the size of neurons in V1 of primates with different sized primary visual corticies, there is a dramatic parallel in the granular PFC. Thus, presently available data suggests that the different trends observed here in V1 of rodents and primates is not attributable to phylogeny, or if they are, there is no one rule to fit all cortical areas. Further data are required in other homologous cortical regions in species of both Orders to provide further clarify.

As each spine receives at least one excitatory input (DeFelipe et al., 1988; Petralia et al., 1994a,b,c; Arellano et al., 2007), the different trends between mammalian orders likely reflect different patterns of connectivity, circuit complexity, functional capacity, and behavioral outcomes (see Nieuwenhuys, 1994; Kaas, 2000; Gould, 2002; Chklovskii et al., 2004; Manger, 2005a; Elston, 2007; Spruston, 2008, for reviews). In future it will be worthwhile to study pyramidal cells in V1 of large brained rodents such as Paca and Capybara, and quantify neuron density (e.g., Collins et al., 2010; Herculano-Houzel et al., 2011; Ribeiro et al., 2013; Young et al., 2013) to provide further bases for comparison (see Manger et al., 2008, for a review). It will also be interesting to compare spine morphologies among species to provide more information of functional specializations between the two orders (e.g., Benavides-Piccione et al., 2003).

The observed differences in pyramidal cell structure detailed here between primates and rodents may also reflect a difference in the processing of visual information in V1 of the different species. For example, the tangential area occupied by the “average” dendritic tree of layer III pyramidal cells in V1 of the adult cane rat is nearly three times the size of that in the adult macaque monkey (Figure 6). Differences in the size, branching structure and spine density result in a more than 10-fold difference in the estimate of the total number of spines, putative excitatory inputs, in the basal dendritic tree of the “average” layer III pyramidal cell between the two species. As V1 in the adult macaque is, on average, more than 40 times the size of V1 in the adult cane rat., the dendritic trees of cells in the adult cane rat sample a portion of the visuotopic field approximately 120 times larger than do those at the same eccentricity in the adult monkey (i.e., the dendritic trees of cells in the cane rat are three times larger than those in the macaque and sample from a visuotopic representation 40 times smaller than do those in the macaque). Thus, 120 cells are required in the macaque monkey V1 to achieve the spatial coverage of a single cell in the cane rat (Figure 6). One hundred and twenty cells in the adult macaque V1 have, on average, a sum total of more than 88,000 spines (120 × 743), being more than 110 times the number of spines in an individual cell in the adult cane rat. Although this is a fairly simplistic calculation, and doesn't take into account features such as branching density (space filling), neuron density, and reciprocal connectivity, it does, we hope, reveal that cortical circuitry in V1 of the cane rat is quantifiably different to that in the macaque monkey. The data suggest high fidelity, high acquity processing in V1 of the macaque relative to that of the rat.

Furthermore, in primates it is well known that several, 35 or more, topographically organized visual cortex areas are present (Felleman and Van Essen, 1991). In rodents the number of visual cortical areas appears to be far less, but the exact number of cortical areas in rodents is a matter of ongoing debate, with arguments for between 3 and 11 areas being forwarded (e.g., Montero, 1993; Rumberger et al., 2001). Despite the contention as to the number of visual areas in rodents, it is clear that there are significantly fewer visual cortical areas in rodents than primates. One may speculate that the task of receiving and integrating visual information in primates may be done in more cortical areas than in rodents, with areas comprising increasing circuit complexity allowing for specialized integration of different visual input. Accordingly, by virtue of the fewer visual areas in the rodent, the neurons in V1 undertake a greater computational task than in the primates, therefore requiring greater sampling of the visual input as evidenced by the larger basal dendritic trees, and more spines, and more local processing within the highly branched dendritic trees. However, this idea requires further testing given the potential number of confounds.

The present physiological data suggests that single unit responses to visual stimuli in rodents are not that different to those in primates (Tiao and Blakemore, 1976; Mangini and Pearlman, 1980; Metin et al., 1988; Girman et al., 1999; Schuett et al., 2002; Van Hooser et al., 2005). However, in view of parallels demonstrated between pyramidal cells structure and function in studies in the developing and aging cortex (McCormick and Prince, 1987; Kasper et al., 1994; Metherate and Aramakis, 1999; Zhang, 2004; Oswald and Reyes, 2008), and between different cortical areas (e.g., Amatrudo et al., 2012; see also Funahashi et al., 1993; Miller et al., 1993, 1996), it is parsimonious suspect that differences in pyramidal cell structure in V1 of rodents and primates are paralleled by differences in their electrophysiological properties (see Elston, 2002, 2003a; Jacobs and Scheibel, 2002; Spruston, 2008, for reviews). We hope the present morphological data will inspire new experiments in which a standardized methodological approach is applied to a comparative study of the electrophysiological properties of pyramidal cells, such as resting membrane potentials, membrane time constant, depolarizing sag, duration of individual action potentials, and spike-frequency adaptation, in V1 of rodents and primates. This avenue of research may lead to interesting insights into how very different cerebral cortices may extract similar information from the visual scene, or, alternatively, reveal previously undescribed differences in neuronal response properties in V1 in rodents and primates.

## Conclusions

Pyramidal cell structure in rodent V1 differs considerably to that in primates. In rodents, pyramidal cells are progressively larger, more branched and more spinous in species with progressively larger brains. In primates, there is relatively little difference in the size, branching structure, and number of spines in pyramidal cells in species with progressively larger brains. The two distinct trends observed in rodents and primates cannot reasonably be attributed a single scaling rule or allometric equation, but appear to reflect fundamentally different modes of cortical organization. As more data becomes available it is becoming increasingly clearer that not only should principles of cortical microcircuitry not be generalized across cortical areas within a given species, but they should not be generalized across species for a given cortical area.

### Conflict of interest statement

The authors declare that the research was conducted in the absence of any commercial or financial relationships that could be construed as a potential conflict of interest.
